# U-shaped association between serum uric acid concentration and mortality in hypertrophic cardiomyopathy patients

**DOI:** 10.1080/03009734.2020.1719245

**Published:** 2020-02-04

**Authors:** Ziqiong Wang, Ying Xu, Hang Liao, Xiaoping Chen, Sen He

**Affiliations:** Department of Cardiology, West China Hospital, Sichuan University, Chengdu, China

**Keywords:** Hypertrophic cardiomyopathy, mortality, serum uric acid, U-shaped association

## Abstract

**Background**. No study has examined the effect of low serum uric acid (SUA) concentrations on mortality in hypertrophic cardiomyopathy (HCM) patients. The aim of the present study was to assess the relations between both low and high SUA concentrations and the risk of mortality across the full range of SUA concentrations in a retrospective cohort of HCM patients.

**Methods.** A total of 454 HCM patients were enrolled in the study, and SUA concentrations were measured at baseline. The primary and secondary endpoints were all-cause mortality and HCM-related mortality, respectively. The associations between SUA concentrations and endpoints were analysed.

**Results.** During a median follow-up of 3.8 years, there were 80 (17.6%) all-cause mortality events, and 52 of them (11.5%) were ascribed to HCM-related mortality. Patients with SUA concentrations of 250–350 µmol/L had the lowest all-cause mortality rate (11.8%) and HCM-related mortality rate (5.0%). Both low and high SUA concentrations were associated with increased all-cause and HCM-related mortality. Adjusted HRs were 2.52 (95% CI 1.13–5.61, *p* = 0.024) and 4.86 (95% CI 1.74–13.58, *p* = 0.003) for all-cause mortality and HCM-related mortality in the lowest SUA group (<250 µmol/L) when compared with the reference group (250–350 µmol/L), respectively. The corresponding HRs in the highest SUA group (≥450 µmol/L) were 2.73 (95% CI 1.42–5.23, *p* = 0.003) and 4.14 (95% CI 1.70–10.13, *p* = 0.002), respectively.

**Conclusions.** Both low and high SUA concentrations were significantly associated with increased risk of all-cause mortality and HCM-related mortality, which supported a U-shaped association between SUA concentrations and mortality in HCM patients.

## Introduction

Uric acid is the end product of purine metabolism (1). It has been reported that high serum uric acid (SUA) concentrations are associated with increased risk of cardiovascular diseases and death (2,3). However, the role of SUA in this constellation still remains controversial (4,5). Recently, several studies have also indicated that low SUA concentrations could predict cardiovascular death and all-cause mortality, which supports a J- or U-shaped association between SUA concentrations and mortality (6–9).

Hypertrophic cardiomyopathy (HCM) is a genetic cardiac disease with marked heterogeneity in clinical expression, natural history, and prognosis (10). Some factors have been identified for risk stratification in HCM patients, such as New York Heart Association (NYHA) class, atrial fibrillation (AF), maximal wall thickness (MWT), and left ventricular outflow tract obstruction (LVOTO), etc. (11). In a retrospective cohort study, Zhu et al. illustrated that high SUA concentrations were associated with adverse outcomes in HCM patients (12). However, no longitudinal studies have evaluated the risk of all-cause and HCM-related mortality across the full range of SUA concentrations in HCM patients while considering both low and high SUA concentrations. Therefore, the purpose of the present study was to evaluate the association between both low and high SUA concentrations with all-cause and HCM-related mortality in a cohort of HCM patients.

## Methods

### Study population

This was a retrospective and longitudinal study. A total of 508 patients with a diagnosis of HCM were consecutively enrolled in the study from December 2008 to May 2016 at West China Hospital of Sichuan University (a tertiary referral centre). The diagnosis of HCM was based on the echocardiographic demonstration of an increase in wall thickness of ≥15 mm in any left ventricular myocardial segment, which was not solely explained by abnormal load conditions (13). Nine patients with inherited metabolic disease or syndromic causes of HCM were excluded from the study (cardiac amyloidosis *n* = 5, restrictive cardiomyopathy *n* = 2, dilated cardiomyopathy *n* = 1, myocarditis *n* = 1). We also excluded patients who were lost to follow-up after the first evaluation (*n* = 41), as well as patients with incomplete biochemistry data (*n* = 4). The final sample size consisted of 454 HCM patients. Detailed information about those patients has been reported elsewhere (14,15). The study was approved by the Ethics Committee on Medical Research of West China Hospital of Sichuan University, and performed according to the principles of the Declaration of Helsinki. Due to the retrospective nature of the study, informed consent was waived.

### Data collection

Blood samples were obtained at admission for all patients. SUA concentrations were measured by the uricase method. Estimated glomerular filtration rate (eGFR) was calculated using the four-variable Modification of Diet in Renal Disease study equation: eGFR (mL/min/1.73 m^2^) = 186.3 × (serum creatinine)^−1.154^× age^−0.203^ (× 0.742 if female) (16). Normal kidney function was defined as eGFR ≥ 60 ml/min/1.73 m^2^ (6). All patients underwent standard two-dimensional transthoracic echocardiography examinations by standard techniques (17). The presence of LVOTO was defined as a gradient >30 mmHg at rest. Other data of baseline characteristics were collected from medical records.

### Follow-up and outcomes

Follow-ups were carried out by clinical consultations, medical records, or telephone interviews. The primary endpoint was all-cause mortality, and the secondary endpoint was HCM-related mortality, which included: (1) sudden cardiac death, (2) heart failure-related death, (3) stroke-related death, and (4) perioperative death due to septal myectomy.

### Statistical analysis

Patients were divided into four groups according to concentrations of SUA: <250 µmol/L (*n* = 43), ≥250 µmol/L and <350 µmol/L (*n* = 161), ≥350 µmol/L and <450 µmol/L (*n* = 148), and ≥450 µmol/L (*n* = 102). Descriptive statistics were used to summarise baseline characteristics.

Baseline characteristics among the four groups were analysed by ANOVA for parametric variables, Kruskal–Wallis test for non-parametric variables, and chi-square or Fisher exact tests for categorical variables. A Kaplan–Meier method was used to estimate the survival in each group, and a log-rank test was used for comparisons. To assess the role of SUA as an independent predictor of mortality, Cox proportional hazard regression analysis was used. The lowest mortality incidence group was defined as the reference group. Age and gender were forced into five multivariable models. Other variables entered a model on the basis of clinical relevance and a univariate relation with mortality (*p* < 0.05). For the final model, the predictors were sought using a stepwise backward modelling approach (*p* = 0.05 for inclusion, *p* = 0.10 for exclusion) including all variables from models 1 to 4. The proportional hazard assumption was verified by means of multivariate Cox regressions. Restricted cubic splines were used to further explore the shape of the dose–response relation between SUA concentrations and risk of all-cause mortality and HCM-related mortality. Finally, we assessed the relation between SUA concentrations and mortality in the patients who did not take hydrochlorothiazide and patients with normal kidney function as sensitivity analyses.

All analyses were performed by EmpowerStats software (www.empowerstats.com, X&Y Solutions, Inc., Boston, MA, USA) and SPSS version 25.0 (SPSS Inc., Chicago, IL, USA). All statistical testing was two-sided.

## Results

### Baseline characteristics

The median age was 57.5 (interquartile range: 46.0–67.0) years (Table 1). Male patients accounted for 55.7%. SUA concentrations ranged from 42.0 to 913.0 µmol/L. Patients with higher SUA concentrations had higher male percentage (*p* < 0.001), higher serum creatinine (*p* < 0.001), larger left atrium (LA) and left ventricle sizes (*p* = 0.006 and *p* = 0.001, respectively), but lower eGFR (*p* = 0.046), high-density lipoprotein–cholesterol (*p* < 0.001), and left ventricular ejection fraction (LVEF) (*p* < 0.001). Other variables did not differ between the four groups.

**Table 1.** Baseline characteristics of the study cohort.

Variables	Whole cohort (*n* = 454)	Serum uric acid concentration (µmol/L)				*p* Value
		<250 (*n* = 43)	≥250, <350 (*n* = 161)	≥350, <450 (*n* = 148)	≥450 (*n* = 102)	
Basic information						
Age (y)	57.5 (46.0–67.0)	59.0 ± 15.1	59.0 (46.0–68.0)	56.5±±4.8	52.1 ± 16.6	0.067
Gender (male)	253 (55.7%)	10 (23.3%)	72 (44.7%)	95 (64.2%)	76 (74.5%)	<0.001
FHHCM	42 (9.3%)	2 (4.7%)	15 (9.3%)	15 (10.1%)	10 (9.8%)	0.739
FHSCD	18 (4.0%)	2 (4.7%)	4 (2.5%)	6 (4.1%)	6 (5.9%)	0.579
NYHA III/IV	156 (34.4%)	20 (46.5%)	49 (30.4%)	47 (31.8%)	40 (39.2%)	0.121
Medical history						
Hypertension	141 (31.1%)	9 (20.9%)	46 (28.6%)	51 (34.5%)	35 (34.3%)	0.280
Diabetes	37 (8.1%)	5 (11.6%)	14 (8.7%)	13 (8.8%)	5 (4.9%)	0.517
COPD	29 (6.4%)	6 (14.0%)	11 (6.8%)	5 (3.4%)	7 (6.9%)	0.092
AF	77 (17.0%)	5 (11.6%)	23 (14.3%)	23 (15.5%)	26 (25.5%)	0.067
Medications/devices/procedures						
Aspirin/clopidogrel	98 (21.6%)	10 (23.3%)	29 (18.0%)	37 (25.0%)	22 (21.6%)	0.512
Warfarin	41 (9.0%)	2 (4.7%)	8 (5.0%)	13 (8.8%)	18 (17.6%)	0.004
Statins	123 (27.1%)	10 (23.3%)	37 (23.0%)	48 (32.4%)	28 (27.5%)	0.279
Beta-blockers	325 (71.6%)	29 (67.4%)	109 (67.7%)	117 (79.1%)	70 (68.6%)	0.109
ACEI/ARB	88 (19.4%)	8 (18.6%)	30 (18.6%)	27 (18.2%)	23 (22.5%)	0.837
HCTZ	27 (5.9%)	3 (7.0%)	10 (6.2%)	9 (6.1%)	5 (4.9%)	0.959
ICD/pacemaker	59 (13.0%)	5 (11.6%)	23 (14.3%)	17 (11.5%)	14 (13.7%)	0.393
Obstruction intervention	41 (9.0%)	4 (9.3%)	13 (8.1%)	19 (12.8%)	5 (4.9%)	0.091
Laboratory test						
eGFR (mL/min/1.73 m^2^)	82.8 (68.6–100.7)	94.2 (79.3–110.3)	88.5 (74.5–107.0)	80.0 ± 24.3	74.4 (56.0–91.2)	0.046
Creatinine (µmol/L)	80.6 (67.0–94.7)	63.1 (55.0–75.0)	74.0 ± 16.7	84.6 (74.0–99.2)	94.7 (80.3–115.3)	<0.001
Glucose (mmol/L)	5.4 (4.9–6.5)	5.4 (4.6–6.7)	5.4 (4.7–6.2)	5.4 (5.0–6.3)	5.5 (5.0–6.8)	0.437
Triglycerides (mmol/L)	1.2 (0.9–1.9)	1.2 (0.9–1.4)	1.2 (0.9–1.6)	1.4 (1.0–2.0)	1.2 (0.9–2.1)	0.054
HDL-C (mmol/L)	1.3 (1.0–1.6)	1.3 (1.1–1.5)	1.4 (1.1–1.7)	1.2 (1.0–1.5)	1.1 (1.0–1.5)	<0.001
LDL-C (mmol/L)	2.4 ± 0.8	2.6 ± 0.9	2.4 ± 0.8	2.4 ± 0.7	2.4 ± 0.8	0.597
Echocardiographic data						
LA (mm)	40.0 (35.0–46.0)	39.2 ± 6.6	38.0 (34.0–45.0)	40.4 ± 6.7	42.6 ± 8.0	0.006
LV (mm)	43.0 (40.0–46.0)	40.0 (36.3–43.0)	43.0 (39.3–46.0)	43.5 (40.0–47.0)	44.0 (40.0–49.0)	0.001
MWT (mm)	19.0 (16.0–22.0)	19.1 ± 5.0	19.0 (17.0–22.0)	20.0 (16.3–22.0)	19 (16.0–21.0)	0.309
LVEF (%)	68.0 (63.0–72.0)	69.0 (65.0–73.0)	70.0 (65.0–73.0)	68.0 (63.0–71.0)	65.0 (59.0–71.0)	<0.001
LVOTO	181 (39.9%)	20 (46.5%)	72 (44.7%)	57 (38.5%)	32 (31.4%)	0.165

ACEI: angiotensin-converting-enzyme inhibitor; AF: atrial fibrillation; ARB: angiotensin receptor blocker; COPD: chronic obstructive pulmonary disease; eGFR: estimated glomerular filtration rate; FHHCM: family history of hypertrophic cardiomyopathy; FHSCD: family history of sudden cardiac death; HCM: hypertrophic cardiomyopathy; HCTZ: hydrochlorothiazide; HDL-C: high-density lipoprotein–cholesterol; ICD: implantable cardioverter defibrillator; LA: left atria; LDL-C: low-density lipoprotein–cholesterol; LV: left ventricle; LVEF: left ventricular ejection fraction; LVOTO: left ventricular outflow tract obstruction; MWT: maximal wall thickness; NYHA: New York Heart Association; TG: triglycerides.

### Clinical outcomes

During a median follow-up of 3.8 years (range: 0.1–9.4), there were 80 (17.6%) all-cause mortality events, and 52 (11.5%) were ascribed to HCM-related mortality.

**Figure 1.**
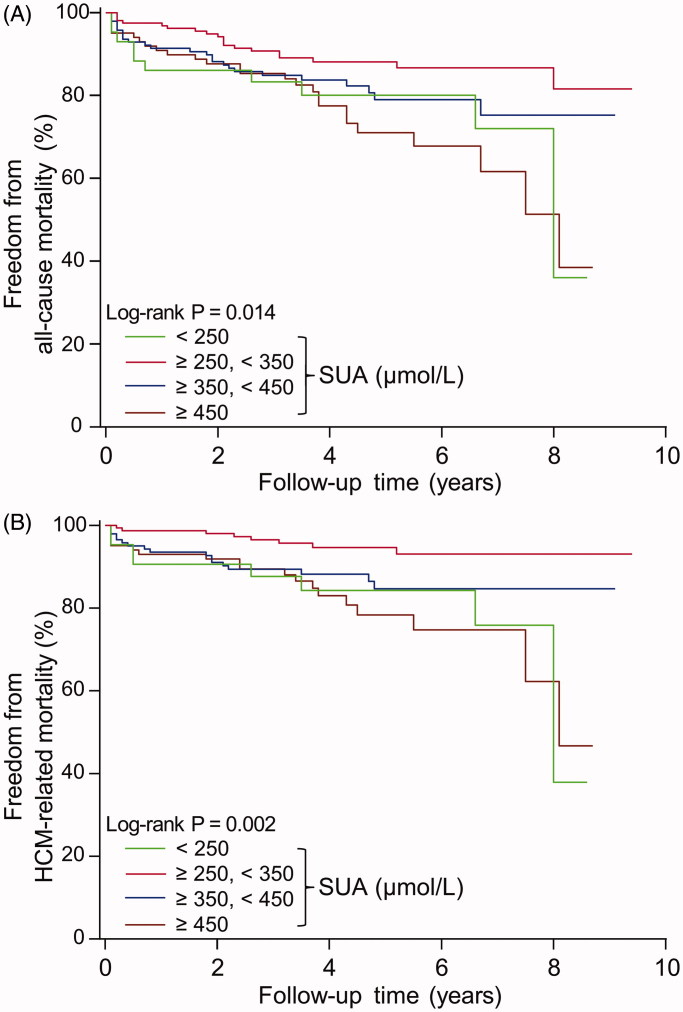
Freedom from all-cause mortality (A) and HCM-related mortality (B) according to different serum uric acid (SUA) concentrations during follow-up period in HCM patients.

Patients with SUA concentrations in the interval 250–350 µmol/L had the lowest mortality rate (Table 2). Likewise, a Kaplan–Meier analysis showed that the survival freedom of all-cause mortality and HCM-related mortality was highest in this group of patients (*p* = 0.014 and 0.002, respectively, Figure 1). A further decrease or increase of SUA was associated with higher risk of mortality.

**Table 2.** Primary and secondary endpoint of the present study.

Endpoints	Whole cohort	Serum uric acid concentration (µmol/L)				
		<250	≥250, <350	≥350, <450	≥450	
No. of patients	454	43	161	148	102	
All-cause mortality						
No. of deaths	80	10	19	25	26	
Mortality rate (%)^a^	17.6	23.3	11.8	16.9	25.5	
HCM-related mortality						
No. of deaths	52	8	8	17	19	
Mortality rate (%)^a^	11.5	18.6	5.0	11.5	18.6	

^a^Binary event rate.

### Relation of SUA to all-cause mortality and HCM-related mortality

Table 3 presents the results of univariate Cox proportional hazard analysis. The lowest SUA group (<250 µmol/L) was found to have an increased risk of all-cause and HCM-related mortality. The corresponding HRs for all-cause and HCM-related mortality, comparing <250 µmol/L SUA with 250–350 µmol/L SUA, were 2.11 (95% confidence interval [CI]: 0.98–4.55, *p* = 0.056) and 3.98 (95% CI: 1.49–10.62, *p* = 0.006), respectively. The highest SUA group (≥450 µmol/L) was significantly associated with increased risk of all-cause and HCM-related mortality. Among the remaining variables, NYHA III/IV, AF, warfarin, serum glucose, and LA were also identified as significant risk factors for both all-cause mortality and HCM-related mortality, while triglycerides, low-density lipoprotein–cholesterol (LDL-C), and LVEF were protective factors.

**Table 3.** Univariate cox proportional hazard analysis for all-cause mortality and HCM-related mortality in HCM patients.

Variables	Change	All-cause mortality HR (95% CI), *p*	HCM-related mortality HR (95% CI), *p*
Age	Per 1-year increase	1.02 (1.01–1.04), 0.006	1.01 (0.99–1.03), 0.219
Gender	Female vs male	1.12 (0.72–1.73), 0.625	1.33 (0.77–2.30), 0.302
FHHCM	Yes vs no	0.71 (0.31–1.63), 0.419	0.94 (0.37–2.36), 0.893
FHSCD	Yes vs no	1.51 (0.61–3.73), 0.376	1.38 (0.43–4.46), 0.582
NYHA III/IV	Yes vs no	2.91 (1.87–4.53), <0.001	2.44 (1.41–4.20), 0.001
Hypertension	Yes vs no	0.81 (0.49–1.33), 0.405	0.76 (0.40–1.42), 0.387
Diabetes	Yes vs no	1.04 (0.48–2.27), 0.913	0.91 (0.33–2.53), 0.861
COPD	Yes vs no	3.18 (1.75–5.76), <0.001	2.13 (0.91–4.99), 0.083
AF	Yes vs no	2.23 (1.39–3.58), 0.001	3.59 (2.07–6.23), <0.001
Warfarin	Yes vs no	2.38 (1.33–4.24), 0.003	3.77 (2.01–7.07), <0.001
HCTZ	Yes vs no	1.09 (0.44–2.71), 0.846	1.01 (0.31–3.23), 0.993
Devices			
None		1	1
Pacemaker		1.55 (0.67–3.57), 0.304	2.49 (1.06–5.86), 0.036
ICD		0.56 (0.20–1.53), 0.257	0.69 (0.21–2.22), 0.529
Procedures			
None		1	1
Alcohol septal ablation		0.48 (0.15–1.53), 0.216	0.25 (0.03–1.77), 0.163
Septal myectomy		1.06 (0.15–7.60), 0.957	1.64 (0.23–11.87), 0.627
eGFR	Per 1 unit increase	0.99 (0.98–0.99), 0.004	0.99 (0.98–1.00), 0.128
Glucose	Per 1 mmol/L increase	1.12 (1.03–1.22), 0.008	1.11 (0.99–1.24), 0.070
Triglycerides	Per 1 mmol/L increase	0.67 (0.49–0.93), 0.015	0.55 (0.35–0.87), 0.010
LDL-C	Per 1 mmol/L increase	0.64 (0.48–0.85), 0.002	0.67 (0.46–0.96), 0.029
LA	Per 1 mm increase	1.04 (1.01–1.07), 0.016	1.06 (1.03–1.10), <0.001
MWT	Per 1 mm increase	1.01 (0.96–1.05), 0.826	0.98 (0.92–1.04), 0.439
EF	Per 1 percent increase	0.97 (0.95–0.99), 0.004	0.96 (0.94–0.98), 0.002
LVOTO	Yes vs no	1.07 (0.68–1.69), 0.757	1.10 (0.63–1.92), 0.749
serum uric acid (µmol/L)			
<250		2.11 (0.98–4.55), 0.056	3.98 (1.49–10.62), 0.006
≥250, <350		1	1
≥350, <450		1.65 (0.91–3.00), 0.098	2.65 (1.14–6.14), 0.023
≥450		2.56 (1.42–4.64), 0.002	4.43 (1.94–10.15), <0.001

Abbreviations as in Table 1.

After adjusting for potential confounding factors, the association between SUA concentrations and endpoints remained consistent. In the final model—after adjusting for age, sex, NYHA III/IV, chronic obstructive pulmonary disease, AF, triglycerides, LDL-C, and LA—HRs for all-cause mortality and HCM-related mortality, comparing ≥450 µmol/L SUA with 250–350 µmol/L SUA, were 2.73 (95% CI: 1.42–5.23, *p* = 0.003) and 4.14 (95% CI: 1.70–10.13, *p* = 0.002), respectively. The corresponding adjusted HRs in SUA <250 µmol/L for all-cause mortality and HCM-related mortality were 2.52 (95% CI: 1.13–5.61, *p* = 0.024) and 4.86 (95% CI: 1.74–13.58, *p* = 0.003) (Table 4).

**Table 4.** Multivariate cox proportional hazard models for all-cause mortality and HCM-related mortality in HCM patients.

Models	serum uric acid concentration (µmol/L)			
	<250	≥250, <350	≥350, <450	≥450
All-cause mortality, HR (95% CI), *p*				
Model 1	1.64 (0.75–3.58), 0.215	1	1.86 (1.01–3.43), 0.047	2.73 (1.47–5.07), 0.001
Model 2	1.58 (0.71–3.50), 0.261	1	1.91 (1.04–3.54), 0.038	2.75 (1.47–5.15), 0.002
Model 3	2.02 (0.93–4.41), 0.076	1	1.73 (0.94–3.20), 0.080	2.59 (1.39–4.84), 0.003
Model 4	2.72 (1.21–6.10), 0.016	1	1.72 (0.92–3.24), 0.091	2.53 (1.26–5.06), 0.009
Model 5	2.52 (1.13–5.61), 0.024	1	1.99 (1.06–3.72), 0.031	2.73 (1.42–5.23), 0.003
HCM-related mortality, HR (95% CI), *p*				
Model 1	3.10 (1.14–8.40), 0.026	1	3.13 (1.33–7.36), 0.009	5.16 (2.19–12.18), <0.001
Model 2	3.16 (1.17–9.06), 0.024	1	3.27 (1.38–7.76), 0.007	4.63 (1.94–11.09), 0.001
Model 3	4.13 (1.52–11.25), 0.005	1	3.03 (1.28–7.18), 0.012	4.20 (1.77–9.96), 0.001
Model 4	4.68 (1.71–12.81), 0.003	1	2.97 (1.24–7.09), 0.014	4.38 (1.77–10.85), 0.001
Model 5	4.86 (1.74–13.58), 0.003	1	3.18 (1.33–7.61), 0.010	4.14 (1.70–10.13), 0.002

Model 1: adjusted for age, sex, FHHCM, FHSCD, NYHA.

Model 2: adjusted for age, sex, hypertension, diabetes, COPD, AF.

Model 3: adjusted for age, sex, warfarin, HCTZ, obstruction intervention and devices.

Model 4: adjusted for age, sex, eGFR, glucose, triglycerides, LDL-C, LA, EF.

Model 5: adjusted for age, sex, NYHA, COPD, AF, TG, LDL-C, LA.

Abbreviations as in Table 1.

### Restricted cubic spline

**Figure 2.**
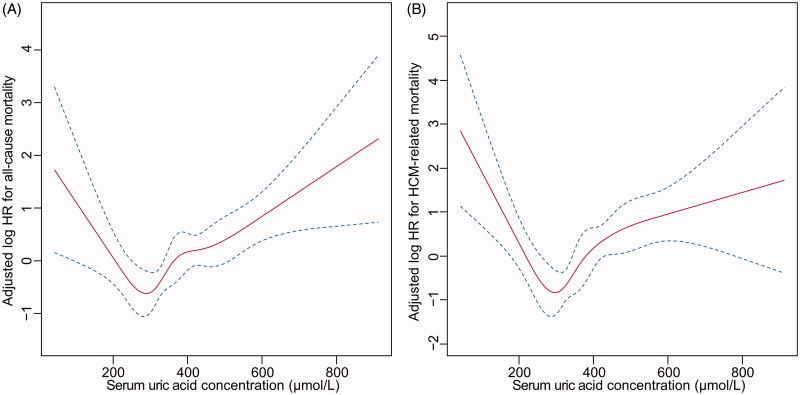
U-shaped association between serum uric acid concentration and all-cause mortality (A) and HCM-related mortality (B).

In the multivariable-adjusted spline (adjusted for the same variables as in model 5), there was a U-shaped association between SUA concentrations and all-cause mortality and HCM-related mortality, with a nadir of risk at SUA around 300 µmol/L. Deviation of SUA from 300 µmol/L was significantly associated with higher mortality risk (Figure 2(A,B)). For SUA concentrations higher than 300 µmol/L, a 10 µmol/L increase of SUA showed a 4.6% (*p* < 0.001) increase of all-cause mortality and 5.2% (*p* = 0.001) increase of HCM-related mortality. For SUA concentrations less than 300 µmol/L, a 10 µmol/L decrease of SUA showed a 9.4% (*p* = 0.03) increase of all-cause mortality and 15.6% (*p* = 0.004) increase of HCM-related mortality.

### Sensitivity analyses

**Figure 3.**
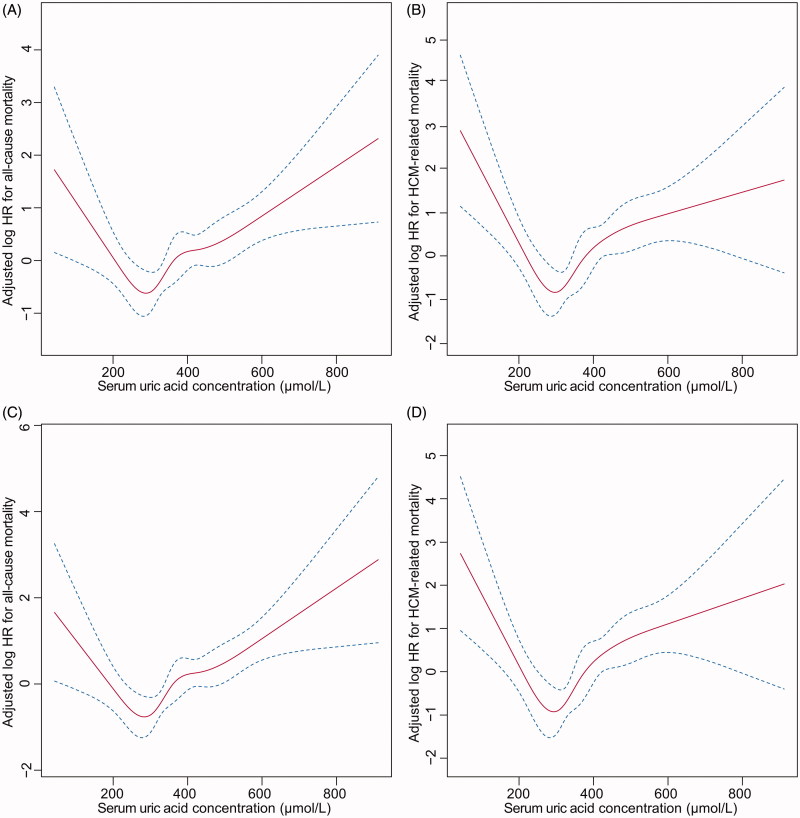
Sensitivity analyses including patients with normal kidney function (A,B) or excluding patients taking hydrochlorothiazide (C,D). U-shaped association between serum uric acid concentration and all-cause mortality (A,C) and HCM-related mortality (B,D).

When including patients with normal kidney function (*n* = 384), there were 56 (14.6%) all-cause mortality and 38 (9.9%) HCM-related mortality events. The adjusted HRs maintained a U-shaped relation, with a nadir of risk at SUA around 300 µmol/L (Figure 3(A,B)). For SUA concentrations higher than 300 µmol/L, a 10 µmol/L increase of SUA was associated with a 2.6% (*p* = 0.213) increase of all-cause mortality and 6.3% (*p* = 0.008) increase of HCM-related mortality. For SUA concentrations less than 300 µmol/L, a 10 µmol/L decrease of SUA was associated with a 11.1% (*p* = 0.026) increase of all-cause mortality and 19.3% (*p* = 0.002) increase of HCM-related mortality.

Upon excluding patients who were taking hydrochlorothiazide, 427 patients remained. There were 75 (17.5%) all-cause mortality and 49 (11.4%) HCM-related mortality events. The association between SUA concentrations and mortality did not change materially (Figure 3(C,D)). For SUA concentrations higher than 300 µmol/L, the risk of all-cause mortality and HCM-related mortality increased 4.5% (*p* = 0.001) and 5.0% (*p* = 0.003) with each 10 µmol/L increase in SUA concentrations, respectively. For SUA less than 300 µmol/L, the risk of all-cause mortality and HCM-related mortality increased 9.9% (*p* = 0.029) and 15.6% (*p* = 0.006) with each 10 µmol/L decrease in SUA concentration, respectively.

## Discussion

In the present study, patients with either low or high SUA concentrations were found to have a higher risk of all-cause mortality and HCM-related mortality. Our study is the first study to reveal a U-shaped association between SUA concentrations and all-cause mortality and HCM-related mortality in HCM patients. The inflection point is approximately 300 µmol/L SUA.

Our findings have some similarities with previous studies but also presented with certain differences (6–9). In a general US population, it was reported that there was a U-shaped association between SUA concentrations and cardiovascular mortality. However, this relation was no longer statistically significant after adjusting for eGFR and albumin–creatinine ratio (ACR) (6). In our study, the association remained stable after adjusting for eGFR. We were unable to examine the effect of ACR due to data unavailability. In another study comprising Korean adults with normal kidney function, Kang et al. found that the overall mortality rate had a U-shaped association with SUA concentrations in males but not in females (7). Furthermore, in a large cohort study of Korean general populations, the authors demonstrated that low SUA concentrations were independently associated with increased risk of all-cause mortality in both genders and increased risk of cardiovascular disease in females only (9). There was no gender-specific relation in our study.

To our knowledge, only one study has been carried out to illustrate the relation between SUA concentrations and all-cause mortality and cardiovascular death in HCM patients (12). In that study, SUA was categorised into tertiles. The adjusted HRs for all-cause mortality and cardiovascular death of subjects in the highest tertile of SUA were 2.33 (95% CI: 1.11–4.89, *p* = 0.025) and 3.10 (95% CI: 1.37–7.04, *p* = 0.007) when compared to that of subjects in the lowest tertile. There was no statistical difference between the second tertile versus the first tertile with regard to the aforementioned outcomes. The cut-off points overlapped but not the same when categorising patients into different groups between that study and our study. By grouping SUA into tertiles, intracategory variations in mortality risk could not be detected, probably leading to a failure of examining the influence of very low and very high SUA concentrations on mortality.

*In vitro* and animal studies have revealed that high SUA concentrations might produce an inflammatory reaction, as evidenced by increased expression of inflammation cytokines, such as interleukin (IL)-6, IL-8, and tumour necrosis factor-α (TNF-α) in endothelial cells. This process was associated with activation of transcription factor NF-κB (18). High SUA concentrations could also stimulate monocyte chemoattractant protein-1 in vascular smooth muscle cells through mitogen-activated protein kinase and cyclooxygenase-2 (19).

A clinical study based on community-dwelling older persons revealed a positive and significant association between SUA concentrations and several inflammatory markers, including neutrophil count, C-reactive protein (CRP), IL-6, IL-18, and TNF-α (20). The above findings supported a role of high SUA concentrations in the process of inflammation. Recently, Wang et al. reported that elevated high-sensitivity CRP was associated with increased risk of adverse outcomes in patients with HCM, suggesting a possible association of an inflammatory state and the clinical progression of HCM (21). Therefore, these inflammatory events induced by SUA might partially explain the poor prognosis of HCM patients with high SUA concentrations in the present study. In addition, the impaired nitro-oxide bioavailability and oxidative stress produced by xanthine oxidase may also be mechanisms behind high SUA-related all-cause and HCM-related mortality (22,23).

The mechanism underlying the increased risk of mortality related to low SUA is not fully understood. SUA acting as an antioxidant may be one of the possible explanations. It has been reported that SUA may exert antioxidant protection against the damage of free radical and reactive oxygen species in ischaemic brain tissue, illustrating the protective effect of SUA for the central nervous system (24). There is also one previous study showing that extremely low SUA concentrations were associated with endothelial dysfunction and vascular damage (25). Therefore, when SUA decreased even more, antioxidant defense might contribute to the relatively high risk of all-cause mortality and HCM-related mortality in HCM patients with low SUA in our study.

This study has several limitations. First, we did not adjust for urate-lowering medications. There were 39.4% of patients diagnosed as having hyperuricaemia (>420 µmol/L for male and >360 µmol/L for female (26)). Some of them might take urate-lowering agents, which in turn would reduce the effect of high SUA on endpoints in our study. However, patients with SUA concentrations less than 250 µmol/L might not be affected by the treatment. And thus the U-shaped association between SUA concentrations and all-cause mortality and HCM-related mortality in HCM patients is credible to some extent. Second, the mortality rate in the present study is higher than in previous studies (11), which might be partially explained by collection bias of patients. All patients were enrolled at the inpatient department of a tertiary referral hospital, and their diseases might be more severe than general HCM populations. Patients with NYHA class ≥3 accounted for 34.1% in the present study. Third, there is a relatively small number of patients (*n* = 43) with SUA level <250 µmol/L in our study. Fourth, some unknown covariates could not be excluded, although extensive adjustment was performed for many important covariates. Fifth, this is a retrospective study from a single centre. Multicentre-based prospective studies are needed to confirm and extend the present findings.
